# Balloon occlusion method using a commercially available ileus tube during endoscopic full-thickness resection: Simple solution to gas leakage

**DOI:** 10.1055/a-2727-5056

**Published:** 2025-11-03

**Authors:** Haruhiro Inoue, Satoshi Abiko, Kei Ushikubo, Kazuki Yamamoto, Yohei Nishikawa, Ippei Tanaka, Naoya Sakamoto

**Affiliations:** 1 Digestive Diseases Center, Showa Medical University Koto Toyosu Hospital, Tokyo, Japan; 2 Department of Gastroenterology and Hepatology, Hokkaido University Hospital, Sapporo, Japan

## Introduction

One of the major challenges in performing endoscopic full-thickness resection (EFTR) for gastric submucosal tumors is leakage of intragastric gas into the abdominal cavity through the full-thickness defect 1 . Previous solutions have ingeniously included handmade balloon devices 2 . However, this approach can be time-consuming, and the handmade nature of the balloon introduces potential risks such as rupture, intraperitoneal dislodgement, and unintentional detachment during the procedure. We report a case in which EFTR was successfully completed while maintaining a stable endoscopic field by using a balloon occlusion method with a commercially available ileus tube (EFTR balloon) inserted into the full-thickness defect.

## Case report

Video showing balloon occlusion method using a commercially available ileus tube during endoscopic full-thickness resection.Video 1

**Fig. 1:**
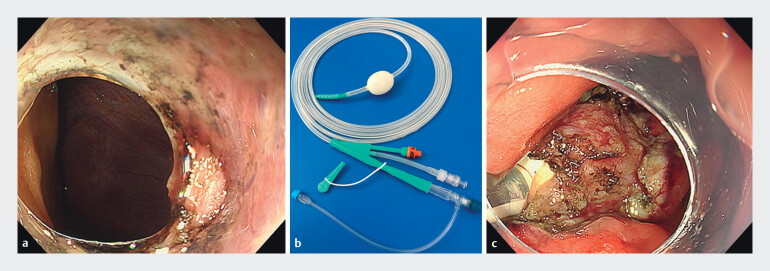
Figure showing balloon occlusion method using a commercially available ileus tube during endoscopic full-thickness resection. **a** Dissection was carefully performed under direct visualization of the serosal layer, resulting in minimal full-thickness resection. Even when we attempted the procedure, the defect tended to enlarge, and the abdominal cavity tissues became visible in the background. **b** A commercially available ileus tube (EFTR balloon: Long Intestinal Tube TYPE CP-II Single balun; Create Medic Co., Ltd. Kanagawa, Japan). **c** An EFTR balloon was then inserted through the defect and inflated within the abdominal cavity and gentle traction on the tube secured its position.

**Fig. 2:**
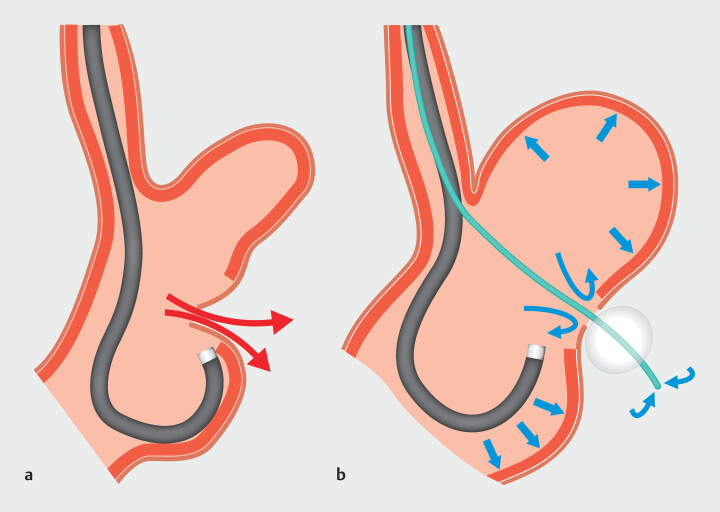
Schema of balloon occlusion method using a commercially available ileus tube. **a** Gas leakage led to collapse of the gastric lumen, thereby hindering continuation of the procedure. **b** The tube was gently pulled to secure the position and this allowed for adequate expansion of the gastric lumen and stable endoscopic visualization.

A 58-year-old man had a progressively enlarging 20-mm submucosal tumor in the greater curvature of the upper gastric body, diagnosed as a fourth-layer gastrointestinal stromal tumor, for which EFTR was performed. After mucosal incision around the lesion, the tumor was grasped and traction was applied. Dissection was carefully performed under direct visualization of the serosal layer, resulting in minimal full-thickness resection. Even when we attempted the procedure, the defect tended to enlarge, and the abdominal cavity tissues became visible in the background ( Fig. 1 **a** ). Tumor resection was performed as promptly as possible. Gas leakage resulted in collapse of the gastric lumen, thereby hindering closure ( Fig. 2 **a** ). Due to increased intra-abdominal pressure, an abdominal puncture was performed. An EFTR balloon ( Fig. 1 **b** ) then was inserted through the defect and inflated within the abdominal cavity. Gentle traction on the tube secured its position ( Fig. 1 **c** ), allowing adequate gastric lumen expansion and stable endoscopic visualization ( Fig. 2 **b** ), and defect closure was performed. By pulling the balloon and placing a stay suture, easy closure near the balloon can be achieved. After deflating and removing the balloon, the defect was completely closed with endoscopic clips ( Video 1 ).
